# P-1239. Systemic Exposure of Olorofim in Children with Invasive Fungal Infections

**DOI:** 10.1093/ofid/ofae631.1421

**Published:** 2025-01-29

**Authors:** Karen Cornelissen, John H Rex, Daniela Zinzi, Roger Bruggemann, James Wright

**Affiliations:** F2G, Manchester, England, United Kingdom; F2G, Limited, WELLESLEY HILLS, MA; F2G, Manchester, England, United Kingdom; Radboud university medical centre, Nijmegen, Gelderland, Netherlands; Wright Dose Ltd, Manchester, England, United Kingdom

## Abstract

**Background:**

Olorofim (OLO) is a novel antifungal active vs. *Aspergillus* (including azole-resistant strains), resistant moulds (e.g., *Lomentospora prolificans [LoPro]*), and dimorphic moulds. Treatment with OLO in children with invasive fungal infection (IFI) is permitted under the Managed Access Program (MAP), where monitoring of trough concentrations is required due to sparsity of data in this population.

**Figure:**
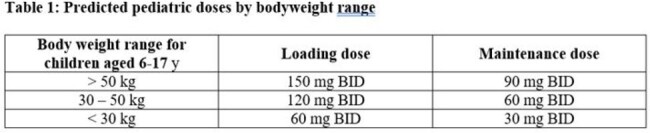

**Methods:**

To be eligible for the MAP, children were aged 6 to 17 years, had an IFI infection that was shown to be or expected to be susceptible to OLO and had limited or no treatment options. Trough plasma samples (collected after an overnight fast) were sent to the central laboratory for analysis of OLO using a validated mass spectrometry assay. As a minimum, samples were taken after at least 7 days dosing and following any dose changes.

The paediatric dose was informed by a population pharmacokinetic (PK) model built using PK data from 52 adult patients with IFI. This model was used to predict systemic exposure in children aged 6 to 17 years based on PK parameters adjusted by weight. Paediatric doses were chosen based on a goal of C_min_ ≥ 0.1µg/mL in over 90% of population, whilst C_max_ and AUC were to be kept as low as possible. A further dose adjustment was required if OLO was given with strong cytochrome P450 3A4 inhibitors or inducers.

**Figure:**
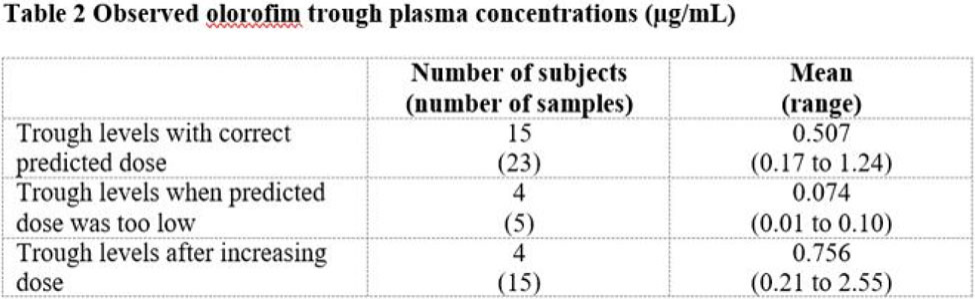

**Results:**

To date, 22 children have received OLO, aged 6 to 17 years (mean = 11.0 y), with body weights of 20 to 80 kg (mean = 37.4 kg). IFIs treated include: IA (7), LoPro (4), *Scedosporium* spp. (4), coccidiomycosis (2), and other rare fungi (5). Trough level data are available for 19 subjects; the predicted maintenance dose resulted in trough levels ≥ 0.1 µg/mL in 15 subjects (79%). For the remaining 4 subjects (21%) OLO dose was increased. After dose adjustment (if required), observed trough plasma concentrations exceeded the threshold needed for efficacy (i.e., > 0.1 µg/mL) on all sampling occasions.

**Conclusion:**

With the relatively small population and sparse PK sampling data available, the predicted body-weight dependent doses for children aged 6 to 17 y are considered reasonable. The dose predictions will be updated as the PopPK model is refined and trough levels will continue to be monitored pending conduct of a formal paediatric study.

**Disclosures:**

**Karen Cornelissen, PhD**, F2G: Stocks/Bonds (Private Company) **John H. Rex, MD**, F2G: Employee **Daniela Zinzi, MD. Infectious Diseases Specialist**, VP Clinical R&D at F2G: Stocks/Bonds (Private Company) **Roger Bruggemann, Pharm, PhD**, F2G: Advisor/Consultant|F2G: Grant/Research Support|Gilead: Advisor/Consultant|Gilead: Grant/Research Support|Mundipharma: Advisor/Consultant|Mundipharma: Grant/Research Support|Pfizer: Advisor/Consultant|Pfizer: Grant/Research Support **James Wright, n/a**, F2G: Advisor/Consultant

